# Effect of organic matter on cyanide removal by illuminated titanium dioxide or zinc oxide nanoparticles

**DOI:** 10.1186/2052-336X-11-23

**Published:** 2013-08-02

**Authors:** Mehrdad Farrokhi, Jae-Kyu Yang, Seung-Mok Lee, Mehdi Shirzad-Siboni

**Affiliations:** 1 Department of Environmental Health Engineering, School of Health, Guilan University of Medical Sciences, Rasht, Iran; 2 Division of General Education, Kwangwoon University, Seoul, Korea; 3 Department of Environmental Engineering, Kwandong University, Gangnung, Korea

**Keywords:** Photocatalysis, Nanoparticle, Titanium dioxide, Zinc oxide, Organic compound, Cyanide

## Abstract

Effect of different type of organic compounds (humic acid, oxalate, ethylenediaminetetraacetic acid, nitrilotriacetic acid, phenol) on the photocatalytic removal of cyanide with TiO_2_ or ZnO was studied in this work with variation of the solution pH, contact time, initial cyanide concentration and type of organic compounds. Photocatalytic oxidation efficiency of cyanide with TiO_2_ was greatly affected by the solution pH. It increased as the solution pH decreased. Also maximum removal of cyanide by ZnO was observed near at neutral pH because of the reduced photocatalytic activity of ZnO at exceedingly low and high pH values originated from either acidic/photochemical corrosion of the catalyst and/or surface passivation with Zn(OH)_2_. Removal efficiency of cyanide greatly decreased in the presence of humic acid, ethylenediaminetetraacetic acid, nitrilotriacetic acid compared to that without presence of organic compound because of the competitive oxidation as well as surface blocking by relatively large organic compounds. The oxidation pattern of cyanide was better described by first-order kinetic model. Finally photocatalytic reaction with TiO_2_ or ZnO can be effectively applied to treat synthetic wastewater contaminated with cyanide.

## Background

Cyanide (CN) is a highly toxic component, which is generated from several industrial activities such as gas production, pharmaceutical, mining, electroplating processes and coal gasification [1,2]. It is known that wastewater generated from electroplating industry contains high concentration of cyanide [3]. Cyanides emissions are increasing at a fast rate and cause a serious concern since they are toxic to living organisms even at very low concentrations [1,4]. Physicochemical methods such as chlorination, electrolytic oxidation, ozonation, etc. and biological methods have been applied to remove cyanides. The generally accepted physicochemical technique for the treatment for the industrial waste containing cyanide compounds is alkaline chlorination [5-10]. The first reaction product generated from chlorination is cyanogen chloride (CNCl), a highly toxic gas having limited solubility. The toxicity of CNCl may exceed that of equal concentrations of cyanide. At an alkaline pH, CNCl hydrolyzed into the cyanate ion (CNO^–^), which has only limited toxicity [1,2].

To conquer these problems, advanced oxidation processes (AOP) have been studied and are recommended as talented techniques*.* Overall, AOPs use hydroxyl free radical (HO^.^) as a strong oxidant to destroy inorganic compounds that cannot be oxidized by conventional oxidants such as oxygen, ozone and chlorine*.* The hydroxyl radical can be generated in aqueous solutions using UV/O_3_, UV/H_2_O_2_, Fe(II)/H_2_O_2_ and UV/TiO_2_[11,12]. Among these methods, photocatalytic reaction using UV/TiO_2_ can treat inorganic compounds and heavy metals at the same time through oxidation and adsorption process. Consequently, this method can be used as pre- or post-treatment methods to extra wastewater treatment methods because it is suitable to fit and inexpensive process in the application of wastewater treatment*.* TiO_2_ has been widely used as the form of suspension or as a thin film in water treatment [6,8,13]. Also ZnO has much attention because electron in valence band of ZnO can be excited at room temperature under low excitation energy. The greatest advantage of ZnO is that it absorbs over a larger fraction of the solar spectrum than that of TiO_2_[11]. The surfaces of ZnO support strong chemisorption of oxygen and are sensitive to ultraviolet (UV) light [14,15]. The most important properties of ZnO are non-toxic in itself, providing attractive photocatalytic efficiency [16]. Photocatalytic reaction using ZnO/UV can simultaneously treat organic compounds and metallic elements in addition to change non-biodegradable to biodegradable organic compounds [17,18]. ZnO is used as an effective, inexpensive and nontoxic photocatalyst for the degradation of synthetic dyes, oxidation cyanide and reduction of Cr(VI) [17,19,20].

As wastewater generated from electroplating industry generally contains high concentration of cyanide, synthetic wastewater containing cyanide concentration ranging from 50 to 200 mg/L was used in this work [3]. The present study investigated the effect of different type of organic compounds (humic acid (HA), oxalate, ethylenediaminetetraacetic acid (EDTA), nitrolotriacetic acid (NTA), phenol) on the photocatalytic removal of cyanide by illuminated titanium dioxide or zinc oxide nanoparticles with variation of solution pH, contact time and initial cyanide concentration. In addition, kinetic parameters were obtained by application of zero, first and second-order equations.

## Material and methods

P-25 TiO_2_ (an 80/20 mixture of anatase and rutile) was obtained from Degussa Corp. It has approximately spherical and non porous shape with greater than 99.5% purity. The specific surface area of the TiO_2_ particles was 50±15 m^2^/g according to Evonik-Industrial Co. Average particle size of the TiO_2_ particles was 21 nm. Also ZnO with greater than 99% purity was obtained from Nano Pars Lima Company in Iran. It has approximately spherical and non porous shape. Average particle size of the ZnO particles was 20 nm. Physicochemical properties of TiO_2_ and ZnO were summarized in Table 1. Also organic compounds with greater than 99% purity were obtained from Merck Company in Germany. All chemicals were analytical grade and solutions were prepared with deionized water (18 MΩ-cm) from a Hydro-Service reverse osmosis/ion exchange apparatus.

**Table 1:** **Physicochemical properties of TiO**_**2 **_**and ZnO**

**Properties**	**TiO**_**2**_	**ZnO**
Specific surface area (BET) (m^2^/g)	50 ± 15	90
Average primary particle size (nm)	21	20
Ignition loss 2 hours at 1000°C, based on material dried for 2 hours at 105°C (wt.%)	≤ 2	≤ 2

### Experimental set-up

**Figure 1:**
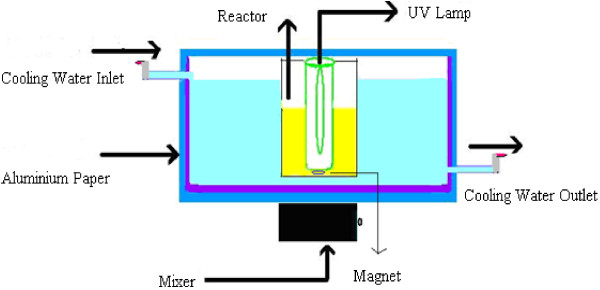
A schematic diagram of the experimental set-up for photocatalysis.

The experimental set-up for photocatalytic reactor used in this work is schematically shown in Figure 1. A 125 W medium-pressure UV-lamp emitting maximum wavelength at 247.3 nm and light intensity equal to 1020 μw/cm^2^ measured by a Spectroline model DRC-100× digital radiometer combining with a DIX-365 radiation sensor (Shokofan Tosee company in Iran). The reactor consists of two compartments: the outer one can contain 2 L solution and the inner one house a UV lamp. The outer part of the reactor is a 10 L water-bath maintained at 25°C for all experiments. All photocatalytic experiments were performed with 1,000 mL solution. During experiments, the test solution in the reactor was constantly stirred.

Stock solution (1,000 mg/L) of cyanide was prepared by dissolving NaCN into deionized water. After the solution temperature decreased to room temperature, deionized water was added to the specified scale of the volumetric flask. Experimental solutions of the desired concentrations were prepared by successive dilutions. Photocatalytic experiments were performed with variation of the solution pH (3, 7, 11), contact time (15–120 min) and initial cyanide concentration (50, 100, 200 mg/L) in the presence of different type of organic compounds (each 100 mg/L). HA, oxalate, EDTA, NTA and phenol were used as organic matter. TiO_2_ and ZnO dosage equal to 1 g/L was used for all experiments because this dosage was identified as an optimum dosage from our previous paper [2,17]. The initial solution pH was adjusted by adding 0.1 M NaOH and HCl. All experiments were performed under ambient conditions for 2 h. TiO_2_ or ZnO suspensions were equilibrated in the dark for 30 min for control test in order to evaluate the contribution of cyanide removal through adsorption process. After the equilibration period, the UV-lamp was turned on and a portion of suspension was periodically taken from the reactor. All the samples were covered by aluminum foil to avoid ambient light. All experiments were accomplished at constant temperature (25 ± 2°C). The sample suspensions were centrifuged at 4,000 rpm for 60 and 10 min to remove TiO_2_ or ZnO nanoparticles, respectively, and then analyzed residual cyanide concentration in supernatant. The remaining concentration of cyanide was analyzed by 4500-CN D. Titrimetric Method according to Standard Methods for the Examination of Water and Wastewater [21]. Indicator solution and titrant were p-dimethylaminobenzalrhodanine and silver nitrate (AgNO_3_), respectively. Then residual cyanide concentration was calculated using following equation:

(1) $$\frac{\mathrm{mg}\;CN^{-}}{L}=\frac{\left(A‒B\right)\times 1000}{\text{mL}\;\text{orginal}\;\text{sample}}\times \frac{250}{\text{mL}\;\text{portion}\;\text{used}}$$

where A and B is volume (mL) of standard AgNO_3_ of sample and of blank, respectively.

All experiments were repeated three times and the average values with error percents were reported.

## Results and discussion

### Removal of cyanide at different solution pH

**Figure 2:**
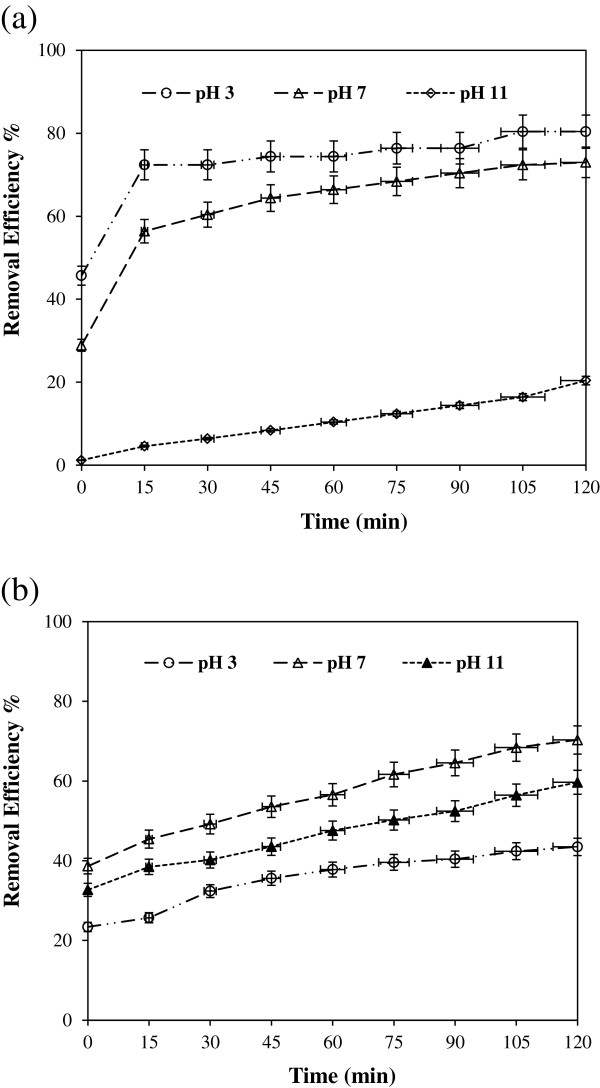
**Effect of solution pH on the removal of cyanide in (a) UV/TiO**_**2 **_**and (b) UV/ZnO system (catalyst dosage = 1 g/L, CN = 100 mg/L).**

Effect of solution pH on the removal of 100 mg/L cyanide by UV/ TiO_2_ and UV/ZnO was investigated at constant catalyst dosage (1 g/L) by varying the initial solution pH (3, 7, 11) at different time interval. Figure 2 shows the removal of cyanide (a) by UV/ TiO_2_ and (b) by UV/ZnO at different solution pH. At initial condition, the removed fraction of cyanide through adsorption process ranged from 28.9% to 45.7% in UV/TiO_2_ system and ranged from 23.4% to 38.7% in UV/ZnO system depending on the solution pH. Cyanide adsorption onto TiO_2_ was greatly dependent on the solution pH compared to ZnO. Figure 2(a) shows the effect of solution pH on the removal of cyanide with UV/TiO_2_. Photocatalytic removal of cyanide increased as the solution pH decreased. The greatest photocatalytic removal of cyanide was observed at pH 3 over the entire reaction time. This result may be explained from combined effects of several factors such as adsorption behavior of cyanide onto metal(hydr-)oxide as well as favorable conditions for photocatalytic reaction at low pH. At high solution pH, increased negative charges on the surface of TiO_2_ causes difficult approach of cyanide ions to the surface of TiO_2_. Similar observations have also been reported previously [22]. Other reasons are an anodic shift of the potential of positive holes and change of the speciation of dissolved CO_2_. As the solution pH increased above neutral pH, bicarbonate is presents as a predominant species. It is a known radical scavenge and causes depression of photocatalytic activity [23].

While highest adsorption of cyanide onto ZnO was observed at pH 7 because ZnO was easily dissoluble in both strong acidic and basic solutions [24]. The effect of pH on the photocatalytic performance can be thus explained in terms of electrostatic interaction between the surface of catalyst and cyanide. Unfortunately, the mere electrostatic argument is unable to exhaustively account for the different photocatalytic efficiency at different solution pH. Other concomitant effect should be considered. It is generally known that ZnO can undergo photocorrosion through self oxidation as expressed in (Eq. 2) [16,18]:

(2) $$\mathrm{ZnO}+{2h}^{+}\to {\mathrm{Zn}}^{2}{}^{+}+1/{2\;O}_{2}$$

In particular, ZnO powder dissolves well at lower solution pH (Eq. 3) [17,19]:

(3) $$\mathrm{ZnO}+{2H}^{+}\to {\mathrm{Zn}}^{2}{}^{+}+H_{2}O$$

In addition, ZnO can undergo dissolution in a strong alkaline environment as expressed in Eq. 4:

(4) $$\mathrm{ZnO}+H_{2}O+{2\mathrm{OH}}^{-}\to \mathrm{Zn}{\left(\mathrm{OH}\right)}_{4}{{}^{2}}^{-}$$

#### Removal of cyanide at different concentrations cyanide

**Figure 3:**
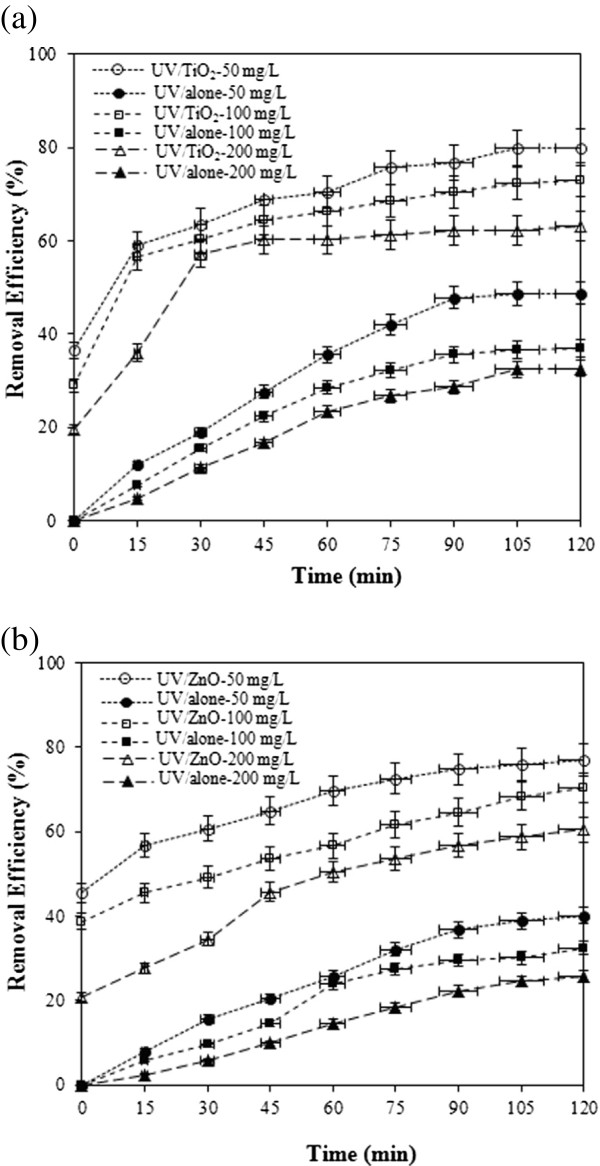
**Effect of initial cyanide concentration on the removal of cyanide in (a) UV/TiO**_**2 **_**and (b) UV/ZnO system (pH=7, catalyst dosage=1 g/L).**

Removal of cyanide by UV/TiO_2_ and UV/ZnO was investigated with variation of initial cyanide concentration (50, 100, 200 mg/L) at initial pH 7 and at constant TiO_2_ and ZnO dosage (1 g/L). Figures 3(a) and 3(b) shows that, for all concentrations, a rapid increase of the cyanide removal was observed up to 30 min and then the slope was gradually reduced. Cyanide removal by UV alone was observed for all concentrations, suggesting photolysis of cyanide. A fraction of cyanide photolysis increased as the cyanide concentration decreased. Percent cyanide removal after 120 min by UV alone in the presence of 50, 100 and 200 mg/L cyanide was 48.7, 37.0 and 32.7%, respectively. Although cyanide removal was possible through photolysis, it was not effective, suggesting necessary of photocatalysts.

In the presence of photocatalysts, it can be seen that percent cyanide removal decreased as the initial cyanide concentration increased. The presumed reason is that when the initial cyanide concentration increased, more cyanide molecules can be removed on the surface of TiO_2_ or ZnO. The large amount of removed cyanide might have an inhibitive effect on the further photocatalytic reaction of cyanide because of the decreased adsorption sites on the TiO_2_ or ZnO as well as the limited oxidants on the surface of TiO_2_ or ZnO [25,26].

The mechanism of cyanide removal is based on the absorption, by the semiconductor particles, of light of energy greater than the band gap energy of the semiconductor, which produces electron hole pairs (e^-^– h^+^). These photogenerated charges can be very reactive depending on the energy of the conduction and valence band edges of the semiconductor and can migrate to the particle surface and react with suitable redox species in solution. From the reaction of cyanide ion with positive hole, CN^–^ is oxidized to OCN^–^, while oxygen is photoreduced to hydrogen peroxide by photogenerated electrons in the conduction band of photocatalysts [20]. This reaction show in equation (5, 6, 7, 8, 9, 10 and 11) [26,27]:

(5) $$\mathrm{ZnO}+h^{\upsilon }\to \mathrm{ZnO}\;\left(e^{-}-h^{+}\right)$$

(6) $$\mathrm{ZnO}\;\left(e^{-}-h^{+}\right)\to \mathrm{ZnO}$$

(7) $$½O_{2}+{2H}_{2}O+{2e}^{-}\to H_{2}O_{2}+O_{2}$$

(8) $${\mathrm{CN}}^{-}+{2\mathrm{OH}}^{-}+{2h}^{+}\to {\mathrm{OCN}}^{-}+H_{2}O$$

(9) $${\mathrm{TiO}}_{2}+h^{\upsilon }\to {\mathrm{TiO}}_{2}\;\left(e^{-}-h^{+}\right)$$

(10) $${\mathrm{CN}}^{-}+{2\mathrm{OH}}^{-}+{2h}^{+}\to {\mathrm{OCN}}^{-}+H_{2}O$$

(11) $$O_{2}+{2H}^{+}+{2e}^{-}\to {2H}_{2}O_{2}$$

#### Effect of type of organic compound

**Figure 4:**
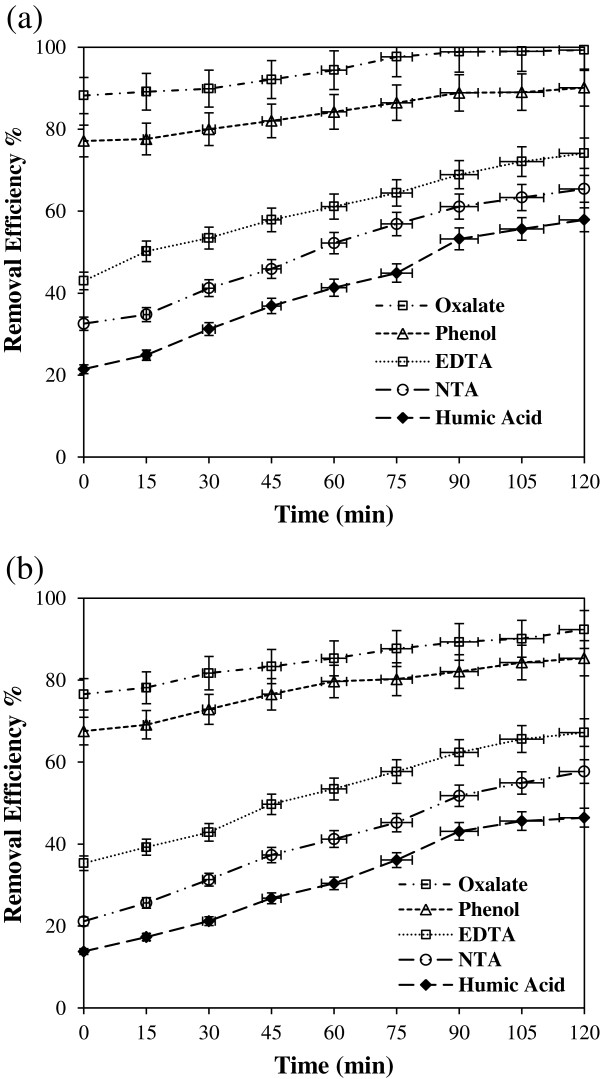
**Effect of different organic compounds on the removal of cyanide in (a) UV/TiO**_**2 **_**and (b) UV/ZnO system (pH=7, catalyst dosage=1 g/L, CN =100 mg/L, organic compound = 100 mg/L).**

Effect of organic compound on the removal efficiency of cyanide by UV/TiO_2_ and UV/ZnO was investigated in the presence of four different organic compounds. Figures 4(a) and 4(b) show that percent removal of cyanide at initial reaction is oxalate > phenol ~ EDTA ~ NTA~ HA. The removed amount of cyanide at initial reaction time in the presence of oxalate and phenol was greater than that in the absence of any organic additives shown in Figure 4(a) and 4(b).

The removed amount of cyanide in the presence of oxalate and phenol gradually increased and was greater than that in the absence of any organic additives over the entire reaction time. Removal efficiency of 100 mg/L cyanide without presence of organic compound increased from 28.9% at 15 min to 73% at 120 min in UV/TiO_2_ system and increased from 38.67% at 15 min to 70.32% at 120 min in UV/ZnO system. The removal efficiency of cyanide in the presence of oxalate increased from 88.2% at 15 min to 99.3% at 120 min in UV/TiO_2_ system and increased from 76.6% at 15 min to 92.3% at 120 min in UV/ZnO system, showing greatly different removal efficiency over the entire reaction time.

The residual cyanide concentration after 120 min of photocatalytic reaction with illuminated TiO_2_ in the presence of oxalate was below the discharge standard of cyanide in USA (1.2 ppm as a maximum value in a day) or Korea (1.0 ppm). It was difficult to explain the enhanced cyanide removal in the presence of oxalate and phenol. Removal efficiency of cyanide greatly decreased in the presence of HA, EDTA and NTA compared to that without presence of organic compound because of the competitive oxidation as well as surface blocking by relatively large organic compounds. Osathaphan et al. (2008) investigated kinetics of the photocatalytic oxidation of cyanide in aqueous TiO_2_ suspensions in the presence of EDTA (0.4-40 mM) at pH 13.0 [28]. They reported a retardation of the cyanide removal rate in the presence of EDTA, indicating that EDTA successfully competed with cyanide for oxidizing species during the photocatalytic processes.

#### Kinetic study

**Figure 5:**
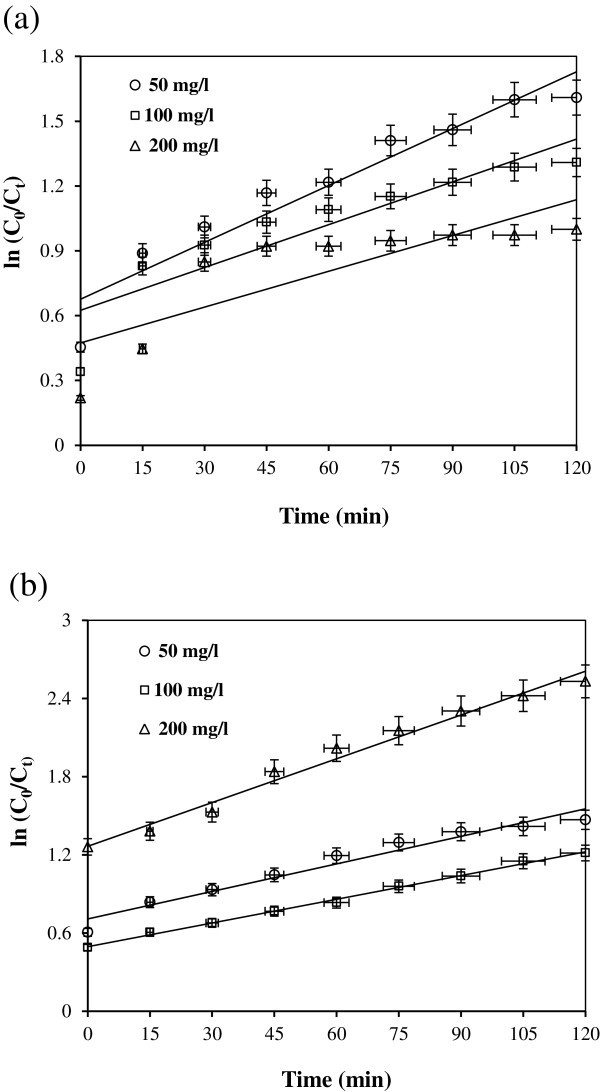
**First-order kinetic model for cyanide removal by (a) UV/TiO**_**2 **_**and (b) UV/ZnO system (pH=7, catalyst dosage=1 g/L).**

**Figure 6:**
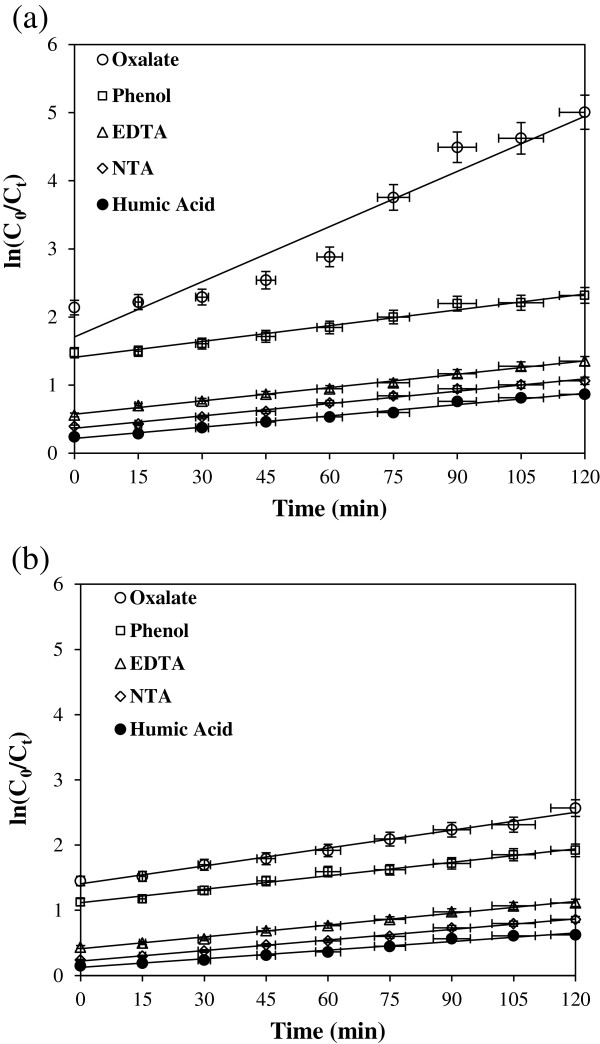
**First-order kinetic model for cyanide removal by (a) UV/TiO**_**2 **_**and (b) UV/ZnO system (pH=7, catalyst dosage=1 g/L).**

In order to obtain the kinetic information, the experimental results in Figures 3(a) and 3(b) and 4(a) and 4(b) were fitted with zero, first and second-order equations. From this evaluation, the removal pattern was better described by first-order kinetic model. Because the initial concentration of cyanide employed in this study was low, the adsorption kinetics can be described adequately by a simplified first-order rate equation (Eq. 12).

(12) $$\ln\left[{\mathrm{CN}}^{-}{}_{0}/{\mathrm{CN}}^{-}{}_{t}\right]=k_{1}t$$

where C_0_ and C_t_ is the cyanide concentration at initial and at time t, respectively. k_1_ is the first-order rate constant and t is the reaction time. Figures 5(a) and 5(b) show the plot of ln[C_0_/C_t_] versus of t for the removal of cyanide by UV/TiO_2_ or UV/ZnO. Figures 6(a) and 6(b) show the plot of ln[C_0_/C_t_] versus of t for the removal of cyanide by UV/TiO_2_ or UV/ZnO in the presence of organic compound. Also first-order rate constants including zero- and second-order rate constants at three different concentrations of cyanide with or without presence of organic compounds are summarized in Tables 2 and 3, respectively. As the cyanide concentration increased, the rate constant in both system decreased.

**Table 2:** **Kinetic parameters for the photocatalytic removal of cyanide by UV/TiO**_**2 **_**and UV/ZnO at different initial cyanide concentration (catalyst dosage = 1g/L, pH=7)**

**UV/TiO**_**2 **_**CN (mg/L)**	**Zero-order**		**First-order**		**Second-order**	
	**k**_**0 **_**(mol L**^**-1 **^**min**^**-1**^**)**	**R**^**2**^	**k**_**1 **_**(min**^**-1**^**)**	**R**^**2**^	**k**_**2 **_**(L mol**^**-1 **^**min**^**-1**^**)**	**R**^**2**^
50	0.490	0.798	0.008	0.919	0.001	0.978
100	0.276	0.688	0.006	0.815	0.002	0.918
200	0.586	0.623	0.005	0.67	0.001	0.718
**UV/ZnO CN (mg/L)**	**Zero-order**		**First-order**		**Second-order**	
	**k**_**0 **_**(mol L**^**-1 **^**min**^**-1**^**)**	**R**^**2**^	**k**_**1 **_**(min**^**-1**^**)**	**R**^**2**^	**k**_**2 **_**(L mol**^**-1 **^**min**^**-1**^**)**	**R**^**2**^
50	0.122	0.903	0.007	0.959	0.002	0.988
100	0.677	0.927	0.006	0.996	0.002	0.985
200	0.26	0.987	0.011	0.983	0.001	0.983

**Table 3:** **Kinetic parameters for the photocatalytic removal of cyanide by UV/ TiO**_**2 **_**and UV/ZnO with organic compounds (catalyst dosage= 1g/L, pH=7, cyanide= 100 mg/L, organic compounds= 100 mg/L)**

**UV/TiO**_**2**_	**Zero-order**		**First-order**		**Second-order**	
**Organic Compounds**	**k**_**0 **_**(mol L**^**-1 **^**min**^**-1**^**)**	**R**^**2**^	**k**_**1 **_**(min**^**-1**^**)**	**R**^**2**^	**k**_**2 **_**(L mol**^**-1 **^**min**^**-1**^**)**	**R**^**2**^
Humic Acid	0.322	0.988	0.005	0.988	0.012	0.975
Oxalate	0.108	0.946	0.027	0.931	0.011	0.82
Phenol	0.12	0.976	0.007	0.978	0.011	0.961
EDTA	0.253	0.986	0.006	0.995	0.001	0.979
NTA	0.297	0.98	0.006	0.989	0.002	0.986
**UV/ZnO**	**Zero-order**		**First-order**		**Second-order**	
**Organic Compounds**	**k**_**0 **_**(mol L**^**-1 **^**min**^**-1**^**)**	**R**^**2**^	**k**_**1 **_**(min**^**-1**^**)**	**R**^**2**^	**k**_**2 **_**(L mol**^**-1 **^**min**^**-1**^**)**	**R**^**2**^
Humic Acid	0.298	0.984	0.004	0.981	0.001	0.972
Oxalate	0.131	0.985	0.006	0.987	0.002	0.94
Phenol	0.154	0.965	0.006	0.993	0.011	0.986
EDTA	0.281	0.988	0.009	0.989	0.001	0.093
NTA	0.314	0.992	0.005	0.995	0.002	0.984

## Conclusions

The major findings of this study are as follow:

Removal of cyanide by UV/TiO_2_ or UV/ZnO increased through photocatalytic removal in the presence of organic compounds, especially oxalate. The cyanide removal decreased at higher pH because of the decreased potential difference between the conduction band of TiO_2_ and cyanide and the anionic-type adsorption of cyanide onto the surface of TiO_2_. Maximum cyanide removal was observed near at neutral pH because of the reduced photocatalytic activity of ZnO at exceedingly low and high pH values. Photocatalytic oxidation rate of cyanide was well described by the first-order kinetics. Photocatalytic reaction with UV/TiO_2_ and UV/ZnO in the presence oxalate can be effectively applied to treat industrial wastewater contaminated with cyanide.

## Competing interests

The authors declare that they have no competing interests.

## Authors’ contributions

All authors read and approved the final manuscript.

## References

[B1] Parga JR Valenzquez V Casillas HM Valenzuela JL Cyanide Detoxification of Mining Wastewaters with TiO_2_ Nanoparticles and Its Recovery by Electrocoagulation Chem Eng Tech Ahead of Print:NA 2009 140 1901 1908

[B2] Shirzad Siboni M Samarghandi MR Yang JK Lee SM Photocatalytic removal of cyanide with illuminated TiO_2_ Water Sci Technol 2011 64 1383 1387 10.2166/wst.2011.738 22179633

[B3] Tiwari D Kim HU Choi BJ Lee SM Kwon OH Choi KM Yang JK Ferrate(VI): A green chemical for the oxidation of cyanide in aqueous/waste solutions Journal of Environmental Science and Health Part A 2007 42 803 810 10.1080/10934520701304674 17474007

[B4] Karunakaran C Gomathisankar P Manikandan G Solar photocatalytic detoxification of cyanide by different forms of TiO_2_ Korean Journal of Chemical Engineering 2011 28 1214 1220 10.1007/s11814-010-0503-1

[B5] Barakat MA Adsorption behavior of copper and cyanide ions at TiO_2_-solution interface J Colloid Interface Sci 2005 291 345 352 10.1016/j.jcis.2005.05.047 16024034

[B6] Barakat MA Chen YT Huang CP Removal of toxic cyanide and Cu(II) Ions from water by illuminated TiO_2_ catalyst Appl Catal Environ 2004 53 13 20 10.1016/j.apcatb.2004.05.003

[B7] Aguado J van Grieken R Lopez-Munoz MJ Marugan J Removal of cyanides in wastewater by supported TiO_2_-based photocatalysts Catal Today 2002 75 95 102 10.1016/S0920-5861(02)00049-4

[B8] Jose P Xavier D Photocatalytic Cyanide Oxidation from Aqueous Copper Cyanide Solutions over TiO_2_ and ZnO J Chem Technol Biotechnol 1992 53 93 96

[B9] Bozzi A Guasaquillo I Kiwi J Accelerated removal of cyanides from industrial effluents by supported TiO_2_ photo-catalysts Appl Catal Environ 2004 51 203 211 10.1016/j.apcatb.2004.02.014

[B10] Ahmed MS Attia YA Aerogel materials for photocatalytic detoxification of cyanide wastes in water J Non-Cryst Solids 1995 186 402 407

[B11] Marugan J van Grieken R Cassano AE Alfano OM Scaling-up of slurry reactors for the photocatalytic oxidation of cyanide with TiO_2_ and silica-supported TiO_2_ suspensions Catal Today 2009 144 87 93 10.1016/j.cattod.2008.12.026

[B12] Yang JK Lee SM Farrokhi M Giahi O Shirzad SM Photocatalytic removal of Cr(VI) with illuminated TiO_2_ Desalin Water Treat 2012 46 375 380 10.1080/19443994.2012.677564

[B13] Hidaka H Nakamura T Ishizaka A Tsuchiya M Zhao J Heterogeneous photocatalytic degradation of cyanide on TiO_2_ surfaces Journal of Photochemistry and Photobiology A: Chemistry 1992 66 367 374 10.1016/1010-6030(92)80009-K

[B14] Look DC Recent advances in ZnO materials and devices Materials Science and Engineering: B 2001 80 383 387 10.1016/S0921-5107(00)00604-8

[B15] Selli E De Giorgi A Bidoglio G Humic Acid-Sensitized Photoreduction of Cr(VI) on ZnO Particles Environ Sci Technol 1996 30 598 604 10.1021/es950368+

[B16] Akyol A Bayramoglu M Photocatalytic degradation of Remazol Red F3B using ZnO catalyst J Hazard Mater 2005 124 241 246 10.1016/j.jhazmat.2005.05.006 15961225

[B17] Shirzad Siboni M Samadi MT Yang JK Lee SM Photocatalytic reduction of Cr(VI) and Ni(II) in aqueous solution by synthesized nanoparticle ZnO under ultraviolet light irradiation: a kinetic study Environ Technol 2012 32 1573 1579 22329148 10.1080/09593330.2010.543933

[B18] Shao D Wang X Fan Q Photocatalytic reduction of Cr(VI) to Cr(III) in solution containing ZnO or ZSM-5 zeolite using oxalate as model organic compound in environment Microporous and Mesoporous Materials 2009 117 243 248 10.1016/j.micromeso.2008.06.026

[B19] Daneshvar N Rasoulifard MH Khataee AR Hosseinzadeh F Removal of C.I. Acid Orange 7 from aqueous solution by UV irradiation in the presence of ZnO nanopowder J Hazard Mater 2007 143 95 101 10.1016/j.jhazmat.2006.08.072 17030415

[B20] Domenech J Peral J Removal of toxic cyanide from water by heterogeneous photocatalytic oxidation over ZnO Solar Energy 1988 41 55 59 10.1016/0038-092X(88)90115-6

[B21] American Public Health Association Standard methods for the examination of water and wastewater 2005 21 Washington, DC 1 1368

[B22] Pedraza-Avella JA Acevedo-Peña P Pedraza-Rosas JE Photocatalytic oxidation of cyanide on TiO_2_: An electrochemical approach Catal Today 2008 133–135 611 618

[B23] Burns RA Crittenden JC Hand DW Selzer VH Sutter LL Salman SR Effect of inorganic ions in heterogeneous photocatalysis of TCE J. Environ. Eng. ASCE 1999 125 77 85 10.1061/(ASCE)0733-9372(1999)125:1(77)

[B24] Yang GCC Chan SW Photocatalytic reduction of chromium(VI) in aqueous solution using dye-sensitized nanoscale ZnO under visible light irradiation J. Nanopart. Res 2008 11 1573 230

[B25] Yeber MC Soto C Navarrete J Vidal G 221 R Optimization by factorial design of copper (II) and toxicity removal using a photocatalytic process with TiO_2_ as semiconductor Chem Eng J 2009 152 14 19 10.1016/j.cej.2009.03.021

[B26] Karunakaran C Gomathisankar P Manikandan G Preparation and characterization of antimicrobial Ce-doped ZnO nanoparticles for photocatalytic detoxification of cyanide Mater Chem Phys 2010 123 585 594 10.1016/j.matchemphys.2010.05.019

[B27] Chandan S Rubina C Kavita G Preliminary study on optimization of pH, oxidant and catalyst dose for high COD content: solar parabolic trough collector Iranian Journal of Environmental Health Science & Engineering 2008 10 1 10 10.1186/1735-2746-10-13 PMC3561206 23369352

[B28] Osathaphan K Chucherdwatanasak B Rachdawong P Sharma VK Photocatalytic oxidation of cyanide in aqueous titanium dioxide suspensions: Effect of ethylenediaminetetraacetate Solar Energy 2008 82 1031 1036 10.1016/j.solener.2008.04.007

